# Successful outcome of Langerhans cell histiocytosis complicated by therapy-related myelodysplasia and acute myeloid leukemia: a case report

**DOI:** 10.1186/1757-1626-1-101

**Published:** 2008-08-18

**Authors:** Khalid A Al-Anazi, Abdulrahman Alshehri, Hazza A Al-Zahrani, Fahad I Al-Mohareb, Irfan Maghfoor, Dahish Ajarim

**Affiliations:** 1 Section of Adult Hematology and Hematopoietic, Stem Cell Transplant, King Faisal Cancer Centre, King Faisal Specialist Hospital and Research Centre, P.O. Box: 3345, Riyadh, 11211, Saudi Arabia; 2 Section of Medical Oncology, King Faisal Cancer Centre, King Faisal Specialist, Hospital and Research Centre, P.O. Box: 3345, Riyadh, 11211, Saudi Arabia

## Abstract

**Background:**

Various therapeutic options are available for the management of Langerhans cell histiocytosis. However, treatment administered to control this disease may be complicated by acute leukemia.

**Case presentation:**

A 34 years old male was diagnosed to have Langerhans cell histiocytosis in March 1999. Unfortunately, the cytotoxic chemotherapy and radiotherapy given to control the repeated relapses and exacerbations of the primary disease predisposed him to therapy-induced myelodysplastic syndrome which transformed into acute myeloid leukemia. After achieving complete remission of his leukemia, the patient received an allogeneic hematopoietic stem cell transplant. The allograft was complicated by chronic graft versus host disease that was controlled by various immunosuppressive agents and extracorporal photophoresis.

**Conclusion:**

Management of complicated cases of histiocytosis requires various therapeutic modalities and a multidisciplinary approach. Having complications of therapy eg myelodysplasia or acute leukemia make the outcome more dismal and the management options limited to aggressive forms of treatment. High dose chemotherapy followed by an allograft may be a curative option not only for therapy-related myelodysplasia/acute leukemia, but also for frequently relapsing and poorly controlled Langerhans cell histiocytosis.

## Background

Langerhans cell histiocytosis (LCH), previously known as histiocytosis-X, is an uncommon reactive disorder of unknown pathogenesis, characterised by abnormal proliferation of Langerhan cells (LCs) into different body organs and tissues [1,2]. LCH has a wide range of clinical presentations from single system involvment eg skin or bone to multi-focal disease involving: liver, lungs, bone marrow and central nervous system [1]. Head and neck involvement is commonly encountered and presents a difficult management challenge [2].

Current therapeutic options include: observations; aggressive local therapies eg surgical resection and radiotherapy; non-specific immunosuppression and cytotoxic chemotherapy [1,2]. However, management should be tailored according to the circumstances of individual patients and at times a multidisciplinary approach is necessary [2,3].

## Case presentation

A 34 year old Saudi male was diagnosed to have LCH in Damascus, Syria in March 1999. He presented with 6 months history of an occipital mass causing bony destruction and bilateral cervical lymphadenopathy. After receiving 8 cycles of cylophosphomide, vinblastine and prednisone, he had regression of the occipital mass and disappearance of the cervical lymph nodes. Five months later, the patient presented to the medical oncologists at King Faisal Specialist Hospital and Research Centre (KFSH&RC) in Riyadh with progression of his disease in the form of extensive bony involvement. After receiving 2 courses of vinblastine and prednisone in addition to radiotherapy to skull, right parotid, right femur and pelvis, the disease became under control. In February 2002, the patient developed nodular lung lesions and cervical as well as inguinal lymphadenopathy. After confirming relapse of LCH, he received 8 more cycles of prednisone and etoposide, following which the second complete remission (CR) was achieved. On 29/7/2003; the patient had a localized relapse of his LCH as he presented with a new lesion behind the right ear which subsided after receiving 4 cycles of etoposide and prednisone. On 6/1/2004, this lesion increased in size so 2 more cycles of etoposide and prednisone were administered, following which the lesion disappeared. On 27/7/2004, the patient was found to have the third relapse as a new mass appeared in the right external auditory meatus that disappeared after receiving localized radiotherapy. On 6/9/2004, the patient presented with a localized relapse in the form of a tiny swelling involving the right frontal skull bone. An accidental blunt trauma caused rupture of the lesion which healed with scars. On 28/2/2005; the patient was admitted to the leukemia unit at KFSH&RC with low grade pyrexia and anemic symptoms for 2 weeks. Physical examination revealed: pallor, tiny inguinal lymphadenopathy and 2 small dark fleshy lesions, one on the forehead and one in the groin. The chest was clear and cardiovascular examination revealed no murmurs or added heart sounds. There was no abdominal tenderness or palpable organomegaly and neurological examination revealed no abnormality. Full blood count (FBC) showed: WBC: 16.3 × 10^9^/L, Hb: 54 g/L and PLT: 40 × 10^9^/L. Blood film revealed 21% blast cells and dysplastic changes. Bone marrow biopsy (BMB) showed a cellular marrow with 85% myeloblasts without any cytogenetic abnormablity. The renal and hepatic profiles were all within normal limits. After establishing the diagnoses of: therapy-related myelodysplastic syndrome (MDS) transforming into acute myeloid leukaemia (AML) and minimal residual LCH, the patient was commenced on an ICE induction course of chemotherapy composed of idarubicin, cytosine arabinoside and etoposide. Following this treatment, the patient achieved the first CR of his acute leukemia (AL). Meanwhile, an HLA identical sibling donor for allogeneic hematopoietic stem cell transplant (HSCT) was identified. On admission to the HSCT unit on 23/4/2005, the patient was asymptomatic and his physical examination revealed no new abnormality. Blood counts, renal and hepatic profiles were all within normal limits. A pre-allograft BMB showed no evidence of leukemia. The patient received a conditioning protocol composed of busulphan and cyclophosphamide. He was given fluconozole, acyclovir and bactrim as infection prophylaxis and methtrexate and cyclosporine as graft versus host disease (GVHD) prophylaxis. On 3/5/2005; the patient received his allograft without any complication. In the early post-HSCT period, the patient developed grade I mucositis treated with intravenous (IV) morphine infusion and one febrile neutropenic episode treated empirically with IV cefepime. No cytomegalovirus infection, acute GVHD, venoocclusive disease of the liver or hemorhagic cystitis were encountered. The patient engrafted his leucocytes on day +19 HSCT and his platelets on day +12 HSCT. After having a successful allograft, the patient was discharged on day +25 HSCT on cyclosporine, zantac and prophylactic antimicrobials. Thereafter, the patient had regular follow up at the HSCT out patient clinic. One year post-HSCT; he developed chronic GVHD of skin, nails, mouth, eyes and liver. Initially he was treated with prednisone 1 mg/kg/day but as his chronic GVHD became reactivated 5 months later, mycophenolate mofetil and extracorporal photophoresis were given. After achieving a good response to the measures taken, the immunosuppressive therapy was gradually tapered. Then the patient continued to have his regular follow up at the HSCT clinic and no new complication was encountered.

## Discussion

P. Langerhans first described LCs in the year 1868. LCs may be considered as distinct macrophages that have acquired antigenic differentiation in thymic and epidermal epithelia [4]. The lesions of LCH contain histiocytes similar to epidermal dendritic cells [5]. The identification of LC has been fascilitated by the discovery of specific markers: enzymes eg ATPase and endogenous peroxidase, birbek granules and immunological membrane markers shared with the macrophage-monocyte-histiocyte system [4]. The detection of clonal histiocytes in all forms of LCH indicates that the disease is probably a clonal neoplastic disorder with highly variable biological behaviour. Thus, the genetic mutations that promote clonal expansion of LCs or their precursors may now be identified [5]. Patients with extensive skin and/or multisystem involvement have high serum level of fms-like tyrosine kinase 3 ligand (FLT 3-L) and M-CSF. Therefore, early hematopoietic cytokines such as: FLT 3-L, stem cell factor and M-CSF may be relevant in the pathogensis of LCH and may be considered as novel therapeutic targets [1].

LCH is a rare but rather severe neoplastic disorder characterized by focal or diffuse systemic proliferation of histiocytic cells at various degrees of differentiation in various body systems/organs eg lymph nodes, bone and bone marrow, liver, spleen and lungs [6]. Retrospective studies in patients with LCH have shown: frequent head, neck and bone involvement, with the skull being the most frequently involved location and with predilection for destructive bony lesions. Organ dysfunction was reported in 5–11%, diabetes incipidus in 10–22%, localized disease in 52.4% and multifocal disease in 47.6% of patients [3,6,7]. Theraputic options included: observation, curettage, chemotherapy eg steroids, etoposide and vinblastine, radiotherapy, HSCT and multimodality treatment [3,7]. LCH should be differentiated from: malignant lymphoma, monocytic leukemia, melanoma, lymphogranulomatosis, immunoblastic lymphosarcoma, sinus histiocytosis with massive lymphadenopathy and undifferentiated carciroma [6,7]. Good outcome and at least 3 year survival have been reported in up to 92% of patients. Single system involvement and absence of organ dysfunction are associated with favourable prognosis while multisystem involvement, organ dysfunction and young age are associated with dismal outcome [3,7,8].

In the year 1985, the first HSCT was performed for a patient with malignant histiocytosis (MH) [9]. Thereafter, autologous and allogeneic HSCT have been used in the management of patients with MH and LCH [9-11]. Small numbers of patients were included in the reported studies. Standard and reduced intensity conditioning (RIC) protocols using stem cells from sibling and matched-unrelated donors were used. Total body irradiation (TBI), cytosine arabinoside, etoposide, melphalan, fludarabine, cyclophosphamide, anti-thymocyte globulin and total lymphoid irradiation were used in the conditioning regimens. TBI-based conditioning regimens proved to be effective as they resulted in sustained disease control while RIC-HSCT was shown to be a feasible option in patients with high risk LCH as treatment related mortality was considerably low [9-11].

There is an association between LCH and both types of AL: AML and acute lymphoblastic leukemia (ALL). AL and LCH can either co-exist in the same patient or one of them may precede the other [12-14]. The frequency of LCH and a malignant neoplasm occurring in the same individual may be greater than previously recognized. Therapy administered to control LCH and genetic predisposition may be possible explanations [12].

Therapy-related myelodysplastic syndrome and therapy-related acute leukemia (t-MDS/t-AL) are serious and rather frequent complications of immunosuppressive treatment, cytotoxic chemotherapy and radiotherapy [15]. T-MDS/t-AL were first recognised in the late 1970s and now they account for 10–20% of all cases of MDS and AL. They have been reported in patients with several malignant disorders treated with various cytotoxic chemotherapeutic agents including: etoposide, anthracyclins, alkylating agents, fludarabine and procarbazine [15]. In patients with t-MDS/t-AL, several chromosomal abnormalities have been described including: deletions and monosomies of chromosomes 5 and 7, 11q23, 21q 22, t (15,17), t (8,21) and inversion 16. Two distinct syndromes have been described: (1) t-MDS/t-AL induced by alkylating agents: characterized by an antecedent dysplasia and a long latency period of 5–7 years. (2) t-MDS/t-AL induced by anthracyclins and etoposide: characterized by absence of antecedent dysplasia, presentation with AML, short latency period of 1–3 years and specific chromosomal abnormalities eg 11q23 and 21q22 [15].

There is no standard therapy for patients with t-MDS/t-AL. Treatment can be aggressive with curative intent, particularly for young and fit individuals. Aggressive chemotherapy protocols eg ICE and 3+7 (daunorubicin and cytosine arabinoside) have induced CRs in 20–100% of patients, but short-lived remissions, early relapses and resistance to chemotherapy were frequently encountered. In patients who are unable to withstand curative regimens, low dose chemotherapy is an alternative option and in elderly or infirm patients, supportive care is a legitimate choice [15]. HSCT offers the best chance of cure. Myeloablative HSCT has yielded long term survival in 30% of patients but transplant-related mortality has been reported to reach 49%, while the non-myeloablative HSCT has been associated with: frequent relapses, short survival and GVHD. Autologous HSCT has resulted in short survival and high rates of relapse [15].

The patient presented suffered repeated progressions and frequent relapses of his LCH. The therapy administered to control/cure his primary disease predisposed him to t-MDS which subsequently transformed into AML. As the patient was relatively young and had no comorbidities, he was subjected to high dose chemotherapy to control his AML. Thereafter, he received an allogeneic HSCT in an attempt to cure his AML. The moderately severe chronic GVHD encountered was controlled by various immunosuppressive agents and extracorporal photophoresis. We believe that the successful allogeneic HSCT in addition to the graft versus leukemia effect of the chronic GVHD not only controlled his t-MDS/t-AL, but also had a long lasting effect on his frequently relapsing LCH.

## Conclusion

Adult-onset LCH tends to progress and relapse. Various modalities of treatment can be used and management plan should take into account the circumstances of individual patients. However, immunosupressive and cytotoxic chemotherapy as well as radiotherapy given to control LCH may be complicated by t-MDS/t-AL. Definitive therapy for t-MDS/t-AL includes high dose cytotoxic chemotherapy followed by an allogeneic HSCT.

## Abbreviations

Langerhans cell histiocytosis; myelodysplastic syndrome; acute myeloid leukemia; hematopoietic stem cell transplant; graft versus host disease.

## Competing interests

The authors declare that they have no competing interests.

## Authors' contributions

All authors participated in the management of the patient presented. All authors read and approved the final form of the manuscript.

## Consent

A written informed consent was obtained from the patient for publication of this case report.
